# Discussion of Dr. Downey’s Paper (Informal.)

**Published:** 1907-03

**Authors:** 


DISCUSSION OF DR. DOWNEY'S PAPER (Informal.)

Dr. W. B. Armstrong asked how the knee was covered.

Dr. Downey replied that the knee and ankle were each encased in plaster separately and then the gap was bridged over by the further application of plaster reinforced by brass wire.

Dr .S. T. Barnett asked how the patient rested in bed.

Dr. Downey said that a folded quilt should be placed under the knee for support.

Dr. W. G. Goldsmith desired to know if a cast could be put on straight. He thought the patient should be anesthetized, especially when the fracture was of the thigh.

Dr. S. T. Barnett moved that Dr. Downey be extended the thanks of the society for demonstrating his ingenius table, which would undoubtedly be of great service to the general practitioner. The motion was carried.

Dr. Cyrus Strickler inquired as to the results obtained where no anaesthetic was given, and said that Downey deserved much credit for his excellent table.

Dr. Michael Hoe thought the table a very ingenius piece of mechanism on account of the large number of uses to which it could be placed. Many of his patients come from improperly treated fractures, especially those involving the hip joint where unmobilization is so important. He thought the table shoudl have some arrangement for obtaining traction with abduction.

Dr. W. S. Goldsmith insisted no apparatus could overcome muscle spasm and that fear of giving an anesthetic to do this accounted for many of the bad results. As pulling frequently increased the spasm, he thought it expedient to wait even as long as 24 hours until an anesthetist could be obtained.

Dr. Willis B. Jones agreed with Dr. Goldsmith as to the importance of an anesthetic and said that whether or not one was given in setting fractures, is regarded as a very important point by courts in deciding damage suits.

Dr. Downey (closing) said that he believed it better to give an anaesthetic when possible but occasionally when alone this could not be done, and that he has had a number of patients with good results without it.

---

A submaxillary swelling should not be dismissed as a lyphatic adentitis without studying Wharton's duct on the same side. Massage of pus therefrom would alter that diagnosis.—American Journal of Surgery.

